# X-ray Phase Contrast osteo-articular imaging: a pilot study on cadaveric human hands

**DOI:** 10.1038/s41598-020-58168-3

**Published:** 2020-02-05

**Authors:** Hélène Rougé-Labriet, Sebastien Berujon, Hervé Mathieu, Sylvain Bohic, Barbara Fayard, Jean-Noel Ravey, Yohann Robert, Philippe Gaudin, Emmanuel Brun

**Affiliations:** 1 Novitom SAS, R-D, Grenoble, 38000 France; 2 Inserm UA7 Strobe, Université Grenoble Alpes, Grenoble, 38000 France; 3 ESRF, the European Synchrotron, Grenoble, 38000 France; 4 Université Grenoble Alpes, IRMaGe, Grenoble, 38000 France; 5 Centre Hospitalier Universitaire Grenoble-Alpes, Hopital Sud, Echirolles, 38434 France

**Keywords:** Cartilage, Imaging techniques

## Abstract

X-ray Phase Contrast Imaging (PCI) is an emerging modality whose availability in clinics for mammography and lung imaging is expected to materialize within the coming years. In this study, we evaluate the PCI Computed Tomography (PCI-CT) performances with respect to current conventional imaging modalities in the context of osteo-articular disorders diagnosis. X-ray PCI-CT was performed on 3 cadaveric human hands and wrists using a synchrotron beam. Conventional CT, MRI and Ultrasound were also performed on these three samples using routine procedures as well as research protocols. Six radiologists and rheumatologists independently evaluated qualitatively and semi quantitatively the 3D images’ quality. Medical interpretations were also made from the images. PCI-CT allows the simultaneous visualization of both the high absorbing and the softer tissues. The 6 reader evaluations characterized PCI-CT as a visualization tool with improved performances for all tissue types (significant p-values), which provides sharper outlines and clearer internal structures than images obtained using conventional modalities. The PCI-CT images contain overall more information, especially at smaller scales with for instance more visible micro-calcifications in our chondrocalcinosis case. Despite a reduced number of samples used, this pilot study highlights the possible medical benefits of PCI for osteo-articular disorders evaluation. Although PCI-CT is not yet available in hospitals, the improved visualization capabilities demonstrated so far and the enhanced tissue measurement quality let suggest strong diagnosis benefits for rheumatology in case of a widespread application of PCI.

## Introduction

Medical imaging plays a key role in rheumatology where it is today essential at the time of diagnosing an osteo-articular disorder. Joint and bone diseases are the most prevalent chronic pain syndromes and long-term disabilities with hundreds of millions people affected worldwide^1^. A whole joint can be affected in many ways with, in most of the cases, several factorial causes. Clinical imaging modalities currently encounter limitations for the simultaneous correct depiction of the different tissues constituting a joint. Conventional X-ray absorption-based CT allows a clear visualization of bone tissues but provides a reduced sensitivity to the soft tissues. Changes in the composition of a joint cartilage or soft tissues are usually rather evaluated using Magnetic Resonance Imaging (MRI). Yet, the images rendered by MRI struggle to define properly the bony changes and the micro-calcifications. In parallel, ultrasonography (US) is still confined to a minor role in clinical routine since, albeit being fast and efficient, its low reproducibility shows problematic when diagnosing acute superficial lesions.

Since the seminal work of Roentgen, with the first ever radiograph of a human hand, technological and scientific advances improved tremendously the image sensitivity and resolution achievable with X-rays. Yet, the core mechanism of X-ray imaging has remained unchanged during all this time, relying on the same physical principle: X-ray attenuation. Phase Contrast Imaging (PCI) emerged almost two decades ago and uses in contrast the fact that X-rays are also refracted when passing through matter. The refraction index of a material can be a thousand times greater than its counterpart absorption factor^2^ for light elements. That translates into a much higher image contrast for a soft tissue while keeping its efficiency for bone tissues when compared to the attenuation based method^3,4^.

Two Dimensional and 3D PCI methods appeared first at synchrotrons before for some of them being successfully adapted to laboratory sources^5–7^. Up to now, the applications of PCI onto human organs focused mainly on breast or lung imaging^8^ where encouraging results could be obtained through various PCI optical set-ups. Clinical trials are now in progress at synchrotrons^9^ whilst clinical prototypes based on conventional X-ray sources are also under commissioning for mammography applications^10^. The literature contains now evidence that the details achieved within PCI allows earlier and better breast cancers diagnosis^11^. Several studies already successfully presented advanced PCI modalities for clinical use^12^. Up to now, to the best of our knowledge, the studies investigating clinical transfers of PCI were restricted to 2D planar imaging^12,13^ and none so far evaluated the performances of 3D PCI Computed Tomography. The aim of this study is hence to assess quality of the 3D imaging of human samples.

However, only a very few studies^14–16^ aimed at musculoskeletal imaging applications, and, to the best of our knowledge, only a few^17^ were carried out in a tomography mode on a human knee. This previous proof of concept study was focusing on cartilage detection whilst setting aside a more detailed investigation of the other tissues. Here, we present the first tomographic images of cadaveric human hands and wrists using PCI-CT. The obtained PCI-CT images were compared both qualitatively and quantitatively with images from the current state of the art conventional imaging modalities. Six independent medical readers assessed a first extensive evaluation of X-ray PCI-CT in terms of medical impact achievable for applications regarding osteo-articular disorders.

## Methods

### Samples preparation

Three cadaveric hands and wrists were transferred after death to the anatomical department of our Hospital. Anatomical pieces were extracted from body to science donors. Approval from the local Grenoble Hospital University was granted for this experimental protocol. Following the French regulation, the donors did not have to sign a statement about this specific study but they did express their consent to anonymously give part of their body to science for ethically approved experiments. All experiments were performed in accordance with the relevant guidelines and local regulations. However, to comply with these regulations, the anatomic pieces obtained from donors did not grant us access to the details of the patients’ medical history. The whole anatomical pieces (skin included) were conditioned into 12 cm diameter PMMA cylinders (5 mm thick). The sealed containers were filled with 10% formalin-saline solution for tissue preservation. Before fixation, one of the anatomical pieces was imaged with ultrasound in order to avoid artefacts in the images that could be caused by the formalin. MRI was performed a week after fixation. The PCI-CT session took place two weeks later. Finally, conventional CT was applied another 6 months later. A 10% formalin fixation is known to alter the MRI properties of the sample, which is why the MRI sequences were performed as soon as possible after fixation. The MRI sequences parameters were also adapted accordingly. Concerning the X-ray CTs, we expect that the formalin uniformly changes the density of the tissues and hence does not change drastically the contrast of the X-ray images.

### X-ray phase contrast imaging

A Propagation Based Imaging (PBI) approach was used to image the sample. PBI explores the phase shifts caused by variations in the refractive index and density of materials by capturing alterations in the measured intensities which can be observed through edge enhancement^18–20^. PBI represents in practice the simplest way to implement phase contrast imaging since no extra optics is required. Such experimental simplicity is however balanced by some requirements on the necessary highly coherent and collimated source such as a synchrotron one^21^. For our imaging campaign, the high intensity X-rays were produced by the ESRF medical beamline wiggler source which provides a quasi-collimated 60 keV monochromatic X-ray beam. The cadaver hands were mounted on a scanning sample stage located at approximately 11 m from the detector. Sets of 3600 projections were collected for each 360-degree rotation. An optical system consisting of a scientific CMOS technology based camera coupled to a gadox scintillator and an objective was used to record the X-ray images. The effective pixel size of the system was 23 µm. A total time of 30 min was necessary to collect the whole tomographic data set for one sample. This rather extended time is due to the reduced height of the synchrotron beam (max. 5 mm) which demanded for sequential partial CT imaging of the sample to obtain a full dataset. A standard filtered back projection combined with a phase retrieval algorithm^22^ was used for the volume reconstructions. The reconstruction time for a complete volume was or around 10 min on a machine equipped with a performant GPU.

While the deposited dose was higher than in clinical routine, the values of the mean radiation deposited doses in the wrist was in the order of magnitude of the milliSievert which remains acceptable for clinical applications. More precisely, the delivered dose was calculated as being equal to 1.98 mSv.

### Conventional modalities

The MRI images were obtained on a 3T TX Achieva Philips Medical scanner (Grenoble MRI Facility iRMaGe). Several MRI sequences were employed including clinical standard sequences recommended for bone, cartilage and soft tissue evaluation (respectively proton density, T1 and T2). An additional research-oriented sequence developed for cartilage imaging (3DWATSC) was also applied. Some sequence parameters were adapted to take into account the fixation of the samples. The conventional CT data were acquired on a General Electrics Healthcare optima CT 660 system using the device standard wrist protocols. The ultrasound images were collected with a Toshiba Aplio 500. The acquisition parameters for MRI, US and CT are summarized in Supplementary Table 1.

### Image quality evaluation

The images were semi-quantitatively evaluated by 6 medical doctors from our hospital (2 rheumatologists and 4 radiologists) with long experiences in musculoskeletal disorders evaluation from images. The visual image quality was each time scored using a 4 point Likert scale: 0: undifferentiated from other structures, 1: visible differentiation, blurry outlines and blurry internal structures, 2: visible differentiation, sharp outlines and blurry internal structures, 3: excellent visualization, sharp outlines and clear internal structures.

Additionally, a quantitative image quality evaluation was performed calculating the standard Signal to Noise Ratio (SNR) and Contrast to Noise Ratio (CNR) of the images defined by:

$$SNR=\frac{\overline{{\rm{\mu }}}}{\sigma }\,CNR=\frac{\overline{{\mu }_{1}}-\overline{{\mu }_{2}}}{\sigma }$$

where $\bar{\mu }$ is the average value in a homogeneous zone of low contrast and σ is the standard deviation in the considered zone. µ_1_ and µ_2_ are defined as average values in two distinct homogenous zones. To avoid resolution bias, the SNR and CNR measurements were made on downscaled dataset with comparable final pixel size for all the modalities.

### Medical interpretation

**Figure 1:**
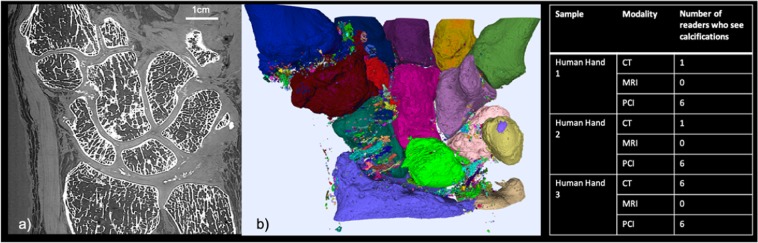
PCI-CT for chondrocalcinosis detection: (**a**) PCI-CT coronal slice from the severe chondrocalcinosis hand case. (**b**) 3D volume rendering of the segmented calcified structures from the PCI volume. The juxtaposed table summarized the number of readers that detected calcifications from each sample dataset.

To simulate the clinical examination conditions, the 6 readers were asked to fill a medical imaging report expounding the possible abnormalities, as they are used to do in clinical routine. The Fig. 1 summarized the number of readers that noted calcification in their report for each set of images (3 series of image for each of the 3 samples).

## Results

### Soft tissues and bone visualization

**Figure 2:**
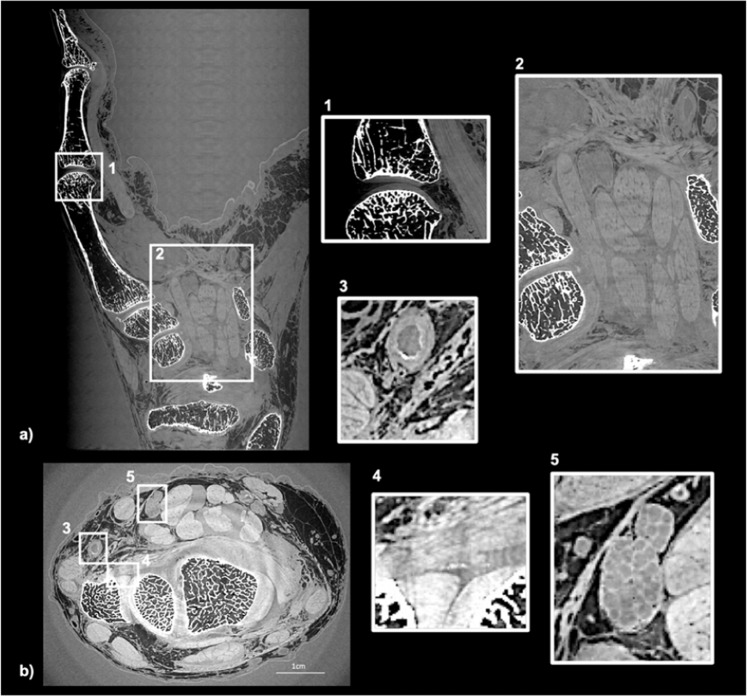
PCI-CT slices of a human hand and wrist. (**a**) Coronal section showing mainly the digitus minimus manus. (**b**) Axial slices in the wrist region. The insets present zoom in the (1) interphalengeal proximal joint, (2)the carpal tunnel, (3) an artery, (4) an enthesis and (5) the median nerve.

From the readers’ perspective, all the different tissues constituting the joint (nerves, muscles, tendons, bones and cartilage) are well rendered at the macroscopic scale in the PCI-CT images. Figure 2 shows PCI-CT coronal (subfigure a) and axial (subfigure b) thin sections of one human hand and wrist with some insets at higher magnification. The readers noticed the particularly good sensitivity of the technique to the hard tissues which allows one to visualize small features such as: bone trabecular structures, appearing geodes, bone demineralization and small atheroma plaques in the lumen of the arteries (Fig. 2 inset 3). Some subtle density changes in the tissue, e.g the small features of denser material in the enthesis of the triangular ligament or a calcification in the joint (Fig. 2 inset 4), were detected by the readers in the PCI-CT images whereas the radiologists were unable to do it with conventional imaging modalities.

The improve image contrast in the soft tissues permits a reliable discrimination. In Fig. 2 inset 1, the cartilage matrix of the distal interphalangeal joint could be well delineated leading to an accurate estimation of the cartilage thickness (mean of 0.76 mm) and a clear distinction between the nerves and tendons (Fig. 2 inset 5). Finally, the high resolution imaging of the nerves through PCI-CT makes possible the counting of the nerve fibers and therefore the detection of a possible pinch.

### Comparison with conventional modalities

**Figure 3:**
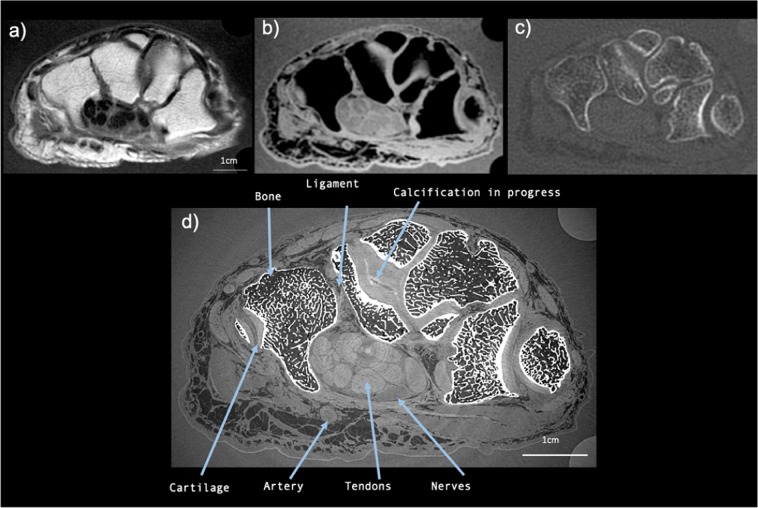
PCI-CT and conventional imaging modalities applied to the same hand area. Images obtained with MRI (**a**) T1W SE, (**d**) (**b**) 3D WATSC, (**c**) CT with bone windowing and (**d**) PCI-CT.

**Figure 4:**
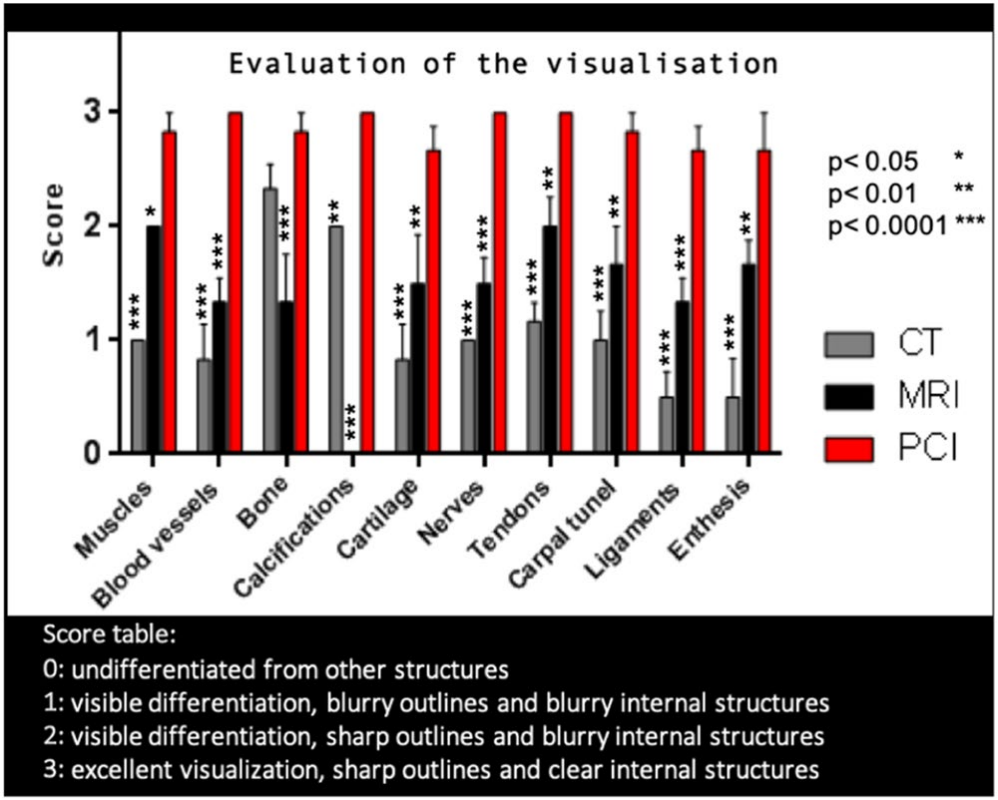
Evaluation of the image quality ranked by radiologists and rheumatologists for the different tissues. The star indicates the p values for the statistical test of PCI-CT vs CT and PCI-CT vs MRI.

The PCI-CT results were compared with images of our three human hands obtained with conventional modalities whose acquisition parameters are summarized in Supplementary Table 1. Figure 3 presents a comparison between axial sections images at the same location obtained with PCI (Fig. 3d) and with the conventional imaging modalities: MRI (Fig. 3a,b), conventional CT (Fig. 3c). The PCI-CT images overall surpass visually the conventional techniques both in terms of contrast and resolution. To validate this observation, we measured global image quality indices that are reported in Table 1. Regarding soft tissue visualization, the Contrast to Noise Ratio values of PCI-CT are approximately improved by a factor 2 when compared to the MRI which is considered today as the clinical gold standard. Such contrast enhancement is confirmed by the Signal to Noise Ratios calculated for bones, muscles, nerves and tendons which proved to be always higher for PCI-CT than the standard modalities. Nevertheless, these standard mathematical metrics are usually poorly suited for a new type of contrast and they rarely directly translate into an aid for a medical interpretation. Therefore, we also performed a semi-quantitative evaluation of the images. Figure 4 displays outcomes of this evaluation made by the experienced rheumatologists and radiologists for the different tissues -Muscles, Blood vessels, Bone, Calcifications, Cartilage, Nerves, Tendons, Carpal tunnel, Ligaments and enthesis. Notwithstanding the readers not being accustomed with phase contrast, all six physicians concluded that the PCI-CT images provide a better visualization of the different tissue types. Figure 4 also shows the results of the statistical tests performed between the image quality evaluations. With p-values under 0.0001 and 0.01 the visualization improvement with PCI is statistically significant for all the components with the exception of bony tissues in CT.

**Table 1:** Signal to noise ratios and contrast to noise ratios measured in different regions of interest.

Modality	Bone	Muscles	Nerves	Tendons	Ligaments
**SNR**					
PCI-CT	14.21	70.6	104.59	67.55	116.21
MRI	1.87	6.45	20.43	8.55	33.83
CT	6.7	2.09	12.04	13.01	7.99
**CNR**					
**Modality**	**muscles/tendons**		**muscles/nerves**		**nerves/tendons**
PCI-CT	33.5		3.87		29.64
MRI	18.3		2.09		16.27

Although ultrasounds (US) being restricted to 2D examination, this technique remains a widespread tool for many osteoarticular disorder diagnosis such as finger tendinous lesions. One radiologist expert in our readers group imaged one hand, using US, in order to evaluate the PCI benefits. Upon examination of the whole sample, one region whose evaluation revealed interest is the index proximal interphalangeal joint where ultrasounds faces limitations. The Supplementary Fig. 1 compares the images of the index proximal interphalangeal joint region obtained by PCI-CT and US. In the PCI-CT case, the tracking in 3D of the tendons’ fibers all along the finger was proved possible with a resolution comparable to what achievable with US.

The Supplementary Fig. 2 presents PCI-CT and conventional 3D images applied to the same hand area with a comparable voxel size. The raw PCI-CT images have been downscaled numerically to obtain 0.3 mm voxel size equivalent images. From these images, one can deduce that the contrast improvement does not originate from a higher resolution but rather from the intrinsic phase contrast signal.

### New accessible joint quality metrics

Thanks to the good sensitivity of PCI-CT within the calcified and cartilage tissues, quantitative measurements of the joint components become feasible providing an invaluable help to grade the different osteoarticular disorders. Figure 1 presents such measurements made on one of our human hand which presented severe chondrocalcinosis. Calcifications can be observed in the coronal section of Fig. 1a whereas a 3D rendering is presented in Fig. 1b where different colors are used to label the various isolated calcified structures. Although the chondrocalcinosis could be diagnosed from the conventional imaging modalities, PCI-CT was able to reveal a significantly higher number of smaller calcifications. Different calcification densities could be noticed from the PCI-CT images but further dedicated investigations are necessary and beyond the scope of the present study for one to be more quantitative and this aspects.

**Figure 5:**
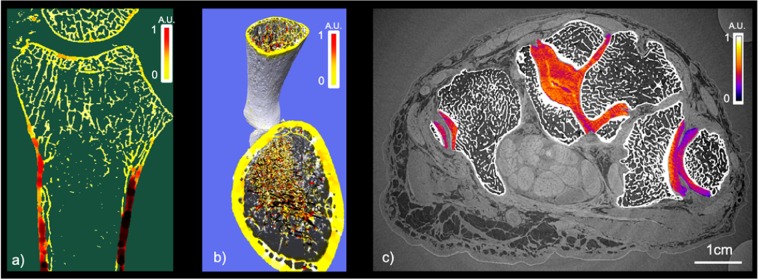
Bone and cartilage quality evaluation: (**a**) Bone quality of an ulna and radius bone: false color representations of the local thickness. (**b**) Estimation of the rode plate distribution in the trabecular bone with false color representation. (**c**) PCI-CT axial slice with a color map of the cartilage density in false color.

The PCI technique provides high sensitivity to calcified structures in addition to specificity for soft tissues. The PCI-CT possibilities were tested on the hard material namely the bone tissue. Figure 5a shows in false color the local thickness of the cortical bone and Fig. 5b renders in 3D the trabecular bone rode/plate structure computed with the iMorph software^23^. Yet, the access denial to the donor medical history for ethical reasons limits in our case the potential of such calculations since no correlation can be inferred.

Finally, Fig. 5c presents a segmentation of the cartilage with the application of a specific colormap relating directly to the density of the material. The image provides a clear visualization of the dense microstructures at the interface between the capitatum and hamatum cartilages. Such finding was similarly encountered in different cartilage interface regions of the 3 samples and a specific investigation is on-going.

### Medical interpretation

Since mathematical metrics or semi-quantitative indices cannot assess solely the medical potential of PCI-CT, the 6 readers were also asked to perform a medical interpretation of the images. The Fig. 4 provides a summary of these interpretations regarding the evaluation of the calcifications. CT is today considered as the standard modality for visualizing calcified tissues. Whilst only one reader noticed the presence of a few calcifications from the non-pathological sample CT dataset, all six readers detected calcified structures from the PCI-CT images. With the hand suffering from chondrocalcinosis, all 6 readers agree that when using PCI-CT, more calcified structures could be detected in various locations (ligaments, intra-articular, cartilage surface and inside the cartilage matrix).

## Discussion and Conclusion

While X-ray PCI is already under translation from large-scale facilities to the clinics for 2D and 3D mammography, this study aimed at evaluating for the first time the potential of PCI-CT in the context of osteo-articular disorders showing slowly developing multifactorial degradations. Despite good knowledge of the possible risk factors, the prevalence of osteo-articular disorders remain nevertheless largely unexplained^24^. Little is really known about the first disorder signs due to the limits of current imaging modalities. This study aimed to evaluate whether PCI-CT can significantly help rheumatologist in their practice. For this study, PCI-CT, MRI and conventional CT with both routine and advanced sequences were assessed by imaging anatomical pieces. All six interrogated experienced radiologists and rheumatologists graded PCI-CT as a more sensitive technique (significant p-values) providing more information on the samples with respect to other imaging modalities. For example, this pilot study shows that 3D PCI permits the detection of more calcifications than conventional CT. In contrast to the mixed readers’ evaluations from the CT images, the opinions converge when using PCI-CT images. Moreover, PCI-CT allows the detection of different densities among the calcifications, constituting a new level of information as already demonstrated for mammography^25^. We speculate that the observed calcifications in the hands come from different degradation processes constituting early signs of musculoskeletal disorders although a full histology remains necessary to validate the hypothesis. In parallel to this bony depiction, the visualization of soft tissues and particularly of nerve inner structures by a non-invasive technique is to our knowledge a first and gives hope, for example, for a reliable evaluation of the carpal tunnel syndromes.

This pilot study encounters limitations from the reduced number of human hand and wrist samples that could be tested as well as the fact that we used a synchrotron facility to produce our images. Such reduced number of samples finds justifications both from the restricted access to facilities worldwide where high-resolution, high-energy phase-contrast imaging can be performed and from the difficulty of transporting cadaver samples to these facilities. Yet, the main goal of this study was to evaluate whether PCI-CT was of sufficient interest to be taken out of synchrotron and to develop dedicated 3D PCI prototypes using conventional X-ray sources. This question arises in the context of articular disorder diagnostics where both hard and soft tissues play a role.

Recent PCI developments let foresee the availability of 2D clinical compatible devices in a near future^26,27^. In this study, the PCI-CT technique used was PBI, which is the simplest one implementable at synchrotron. Nevertheless, recent progresses demonstrated the feasibility of PBI with a less costly source from a laboratory setup, for instance, for the evaluation of human mummy hand^21^. Comparable or better results can also be expected by using alternative phase derivative sensitive PCI techniques such as Grating Interferometry^28^, Analyzer Based Imaging^29^ or Edge Illumination^30^, and these methods are actually considered as more sensitive to certain features^2^. Another more recent technique which is Speckle Based Imaging^31,32^ also let hope for a transfer of 3D PCI to clinical set-up while solving the remaining acquisition time and deposited dose issues. When those 3D clinical setups will be ready, further work will be necessary to extend this study to other osteo-articular pathologies as well as to define the corresponding PCI-CT semiology. The phase signal cannot be retrieved as easily as the attenuation one because no detector is able to directly sense the phase signal and the X-ray refraction angles involved are very small. In the recent decades, several techniques have been developed for recovering phase information. The phase information is not captured directly and several 2D and 3D PCI methods were first developed at synchrotrons, and for some of them, later adapted to laboratory sources with comparable image quality thanks to progress both the X-ray instrumentations and mathematical methods^12,33,34^.

To conclude, PCI-CT provides in a single examination, a better and a more detailed depiction of the different tissues of a whole human joint. The sensitivity of PCI-CT makes possible the evaluation of the pathology stages with probably a good specificity which could be useful as an aid to the follow-up o, but which must be demonstrated later. Albeit MRI and CT are well established for clinical routine evaluation, PCI-CT is still a new and unconventional technique for which radiologists must accustom to. All readers acknowledge that work needs to be done in order to sort the relevant criteria from the PCI information.

## Supplementary information

Supplementary Video1.

Supplementary Video3.

Supplementary Video2.

Supplementary Information.
