# Supplementary material for: X-ray Phase Contrast osteo-articular imaging: a pilot study on cadaveric human hands
Source: Sci Rep. 2020 Feb 5;10:1911. doi: 10.1038/s41598-020-58168-3 (PMC7002527; doi:10.1038/s41598-020-58168-3)
Supplement: Supplementary file 4 — Supplementary Information. [file 41598_2020_58168_MOESM4_ESM.docx]

**X-ray Phase Contrast osteo-articular imaging: a pilot study on cadaveric human hands**

Hélène Rougé-Labriet^1,2^, Sebastien Berujon^3^, Hervé Mathieu^4^, Sylvain Bohic^2,3^, Barbara Fayard, Jean-Noel Ravey^5^, Yohann Robert^5^, Philippe Gaudin^5^ , Emmanuel Brun^2^

^1^Novitom, R-D, Grenoble, F-38000, France

^2^Inserm UA7 Strobe, Université Grenoble Alpes, RSRM, Grenoble, F-38000, France

^3^ESRF, the European Synchrotron, Grenoble, F-38000, France

^4^Université Grenoble Alpes, IRMaGe, Grenoble, F-38000, France

^5^Centre Hospitalier Universitaire Grenoble-Alpes, Hopital Sud, Echirolles, F-38434, France


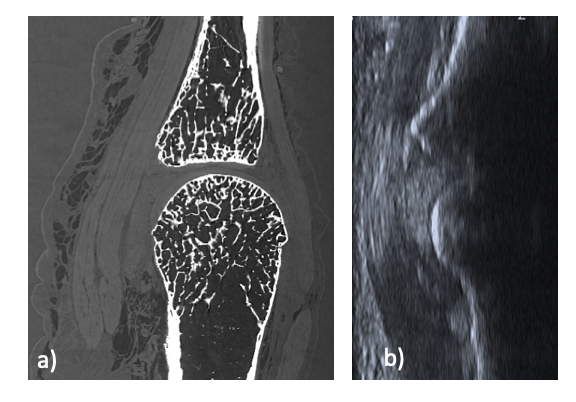


Supplementary figure 1: (a) PCI and (b) conventional Ultra sound images.


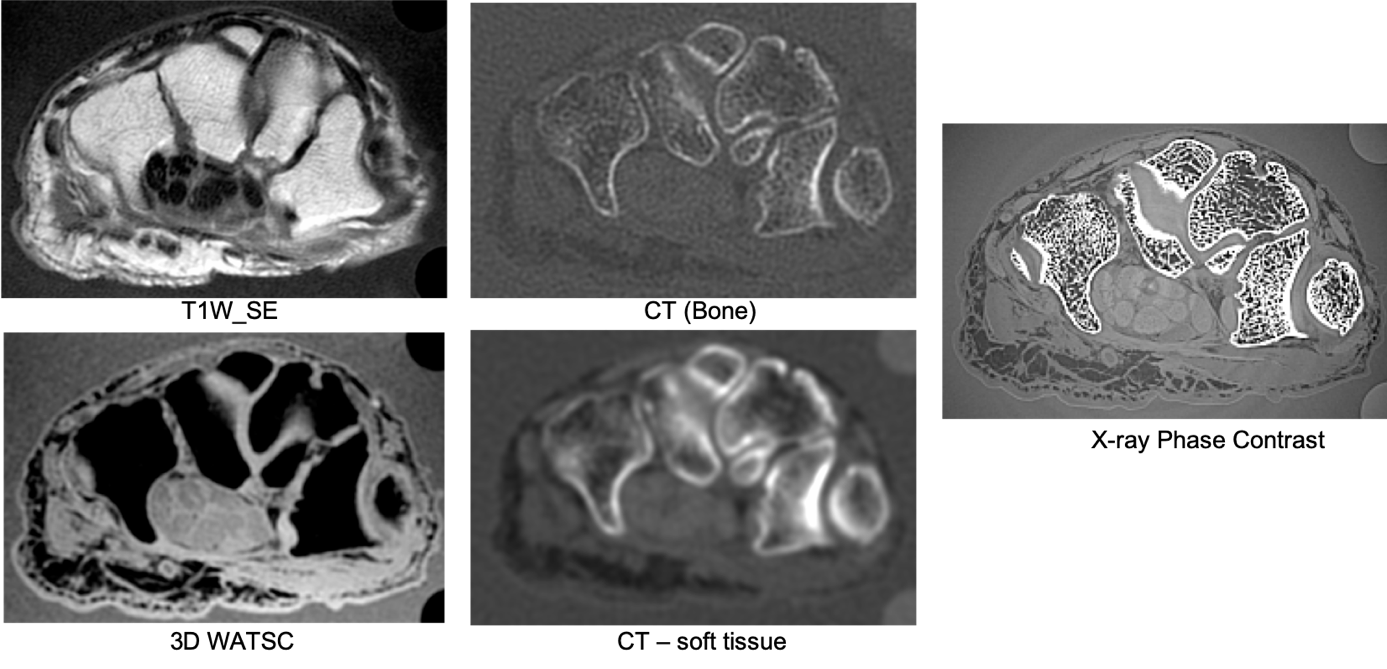


Supplementary figure 2: Slices of the same wirts area obtained using MRI ( T1W_SE, 3D WATSC), CT (bone and soft window) and PCI. All slices have the same voxel size (0.3 mm).

| **MRI sequences** | | | | | |
| --- | --- | --- | --- | --- | --- |
| Machine | 3T Philips Medical System Achieva | | | | |
| Parameter | T1W_SE | T2W_aTSE | PDW | 3D WATSC | T1W_SE_SPIR |
| Repetition time (TR) | 589 ms | 4575 ms | 4490 ms | 20 ms | 570 |
| Echo time (TE) | 18 ms | 100 ms | 30 ms | 5 ms | 18 |
| Flip angle | 90° | 90° | 90° | 90° | 90° |
| Band width | 128 Hz | 356 Hz | 160 Hz | 445 Hz | 128 Hz |
| Slice thickness | 3 mm | 3 mm | 3 mm | 0.62 mm | 3 mm |
| Matrix | 512^2^ | 512^2^ | 512^2^ | 512^2^ | 512^2^ |
| Acquisition time | 2 min 42 s | 2 min 42 s | 2 min 42 s | 2 min 50 s | 2 min 55 s |

| **Phase Contrast CT** | |
| --- | --- |
| Machine | ESRF |
| Energy | 60keV |
| Current | 180mA |
| Resolution | 0.100 mm |
| Dose | 3 mSv |
| Acquisition Time | 5 min |

| **CT acquisitions** | | |
| --- | --- | --- |
| Machine | Ge optima ct 660 | |
| Parameter | standard | HR |
| Energy | 100kvp | 100kvp |
| Current | 100mAs | 120mAs |
| Longitudinal resolution | 0.625mm | 0.100mm |
| Slice thickness | 0.625mm | 0.625mm |
| In-plane resolution | 0.28mm | 0.28mm |
| Acquisition Time | 1.643 s | 1.643 s |
| Dose | 8.89 mGy | 10.3 mGy |

| **UltraSound** | |
| --- | --- |
| Machine | Toshiba Aplio 500 |
| Prode | 10 Mhz |
| FPS | 32 |
| Focusing | 0.9 mm |
| Acquisition Time | 2 min |

*Supplementary Table 1 Acquisition parameters for the PCI-CT, Ultra sound, Conventional CT and MRI data collection.*

|  | ***Human Hand 1*** | | | ***Human Hand 2*** | | | ***Human Hand 3*** | | |
| --- | --- | --- | --- | --- | --- | --- | --- | --- | --- |
| ***Modality*** | *CT* | *MRI* | *PCI* | *CT* | *MRI* | *PCI* | *CT* | *MRI* | *PCI* |
| ***Number of readers who see calcifications*** | *1* | *0* | *6* | *1* | *0* | *6* | *6* | *0* | *6* |

*Supplementary table 2: Number of readers that detected calcifications in the datasets for each sample. Supplementary Video 1: Axial thin sections (100µm thick) sequences of one hand*

*Supplementary Video 2: Close up video of sagittal view of one finger*

*Supplementary Video 3: Coronal thin sections (100µm) sequences of the chondrocalcinosis bearing hand.*
